# Effects of Redox Disturbances on Motility, Contractility and Muscle Tissue Pathogenesis

**DOI:** 10.1155/2019/3272035

**Published:** 2019-06-16

**Authors:** C. Karatzaferi, M. Sandri, G. K. Sakkas, C. Smith

**Affiliations:** ^1^Experimental Physiology & Therapeutic Exercise Lab, DPESS, University of Thessaly, Greece; ^2^Venetian Institute of Molecular Medicine (VIMM), Padova, Italy; ^3^Department of Biomedical Sciences, University of Padova, Padova, Italy; ^4^Department of Physiological Sciences, Stellenbosch University, Stellenbosch, South Africa

Whether in health or disease, reactive oxygen and nitrogen species (ROS/RNS) affect smooth and striated muscle status and function in ways not always discernible or appreciated. Despite the technological and methodological advancements, some key challenges still exist. On the one hand, one challenge is to appreciate acute effects on contractility and/or bioenergetics within a realistic functional context, effectively linking in vitro observations to in vivo conditions. On the other hand, chronic effects on indices of clinical significance are more difficult to clarify given the interplay of redox status variations with systemic inflammation and autophagy but also with lifestyle factors such as nutrition and physical activity—which impact on systemic health indices, as well as directly on smooth and striated muscles.

Redox disturbances are associated with various hallmarks of health deterioration. Regarding skeletal muscle atrophy, a common denominator of many diseases, it remains quite difficult to discern disuse-induced from disease-induced atrophy and the role of ROS-induced signalling (e.g., Malavaki et al.). That is because prolonged periods of contractile inactivity lead to increased production of intramuscular ROS [1], while both immobilization [2] and unloading [3] have been associated with elevated ROS. Many groups are researching the role of ROS/RNS in disturbing the protein balance from protein synthesis to protein degradation via acceleration of proteolysis and depression of synthesis [4]. Others examine if oxidative stress may help explain chronic disease effects on muscle status [5]. Likewise, there is increasing work on the interplay of autophagy, viewed as an antiaging system, and redox imbalances that may affect the contractile and the neuromuscular junction machineries and may modify the overall progress of neurogenic muscle atrophy (e.g., [6, 7]). Moreover, redox imbalances directly modulate force generation with scientific interest turning towards the interaction between muscle adaptations to chronic redox imbalances and acute responses to ROS accumulation (e.g., [8]). Various international groups and networks have thus undertaken the challenge to characterize the crosstalk between other organ systems and muscles and to decipher the role of redox imbalances in early muscle disease detection, or to monitor longitudinal changes, in order to devise preventive strategies to counteract muscle tissue pathogenesis, whether in striated or smooth muscles.

The aim of the present special issue was to provide updated or novel information regarding the role played by redox disturbances in the development of muscle dysfunction acutely and in the long term, stressing the awareness of these concepts for the monitoring, interpretation, and management of disease effects on muscle.

The themes covered in this issue ranged from molecular insights into the role of abnormalities in processes such as ribosome biogenesis in smooth muscle (Q. Wu et al.) and iron homeostasis in cardiomyocytes (A. Bolotta et al.) in redox status to evidence of strategies which may modulate mitochondrial dysfunction and muscle wasting in sepsis (X. Yu et al., C. Andreani et al.). Two descriptive studies characterized skeletal muscle atrophy in chronic diseases (renal insufficiency and rheumatoid arthritis) and demonstrated the inadequacy of frequently used circulating redox markers to reflect muscle redox status. Resting plasma redox status, of the indices studied, did not readily correlate with intramuscular levels (K. P. Poulianiti et al.), while different muscles also exhibited distinct differences in predominating antioxidant mechanisms at play (A. B. Oyenihi et al.), highlighting that tissue-specific research approaches are still much needed. In addition, a review provided a holistic picture of oxidative stress-related mechanisms by which ageing muscle loses both mass and strength (P. Szentesi et al.). This review is particularly timely, given not only the increasing average age and life expectancy of the global population but also the increasing evidence for western lifestyle-related accelerated ageing in which skeletal muscle is greatly affected; as are other muscle types. This was demonstrated in rats fed with a hypercaloric diet, where redox disturbances were linked to impaired intestinal contractility (I. L. L. de Souza et al.). Finally, in a model of experimental ischemia/reperfusion injury in rats (Y. Zhang et al.), the integrated crosstalk between nerve stimulation, inflammatory response, redox signalling, and endothelial function was further elucidated.

All of the studies contained in this issue advance our understanding of the effects that redox imbalances may have on muscle properties and pose new exciting questions to be pursued by the scientific community.
